# Comparative Transcriptomics Analysis of the Symbiotic Germination of *D. officinale* (Orchidaceae) With Emphasis on Plant Cell Wall Modification and Cell Wall-Degrading Enzymes

**DOI:** 10.3389/fpls.2022.880600

**Published:** 2022-05-06

**Authors:** Juan Chen, Yanjing Tang, Annegret Kohler, Annie Lebreton, Yongmei Xing, Dongyu Zhou, Yang Li, Francis M. Martin, Shunxing Guo

**Affiliations:** ^1^Key Laboratory of Bioactive Substances and Resource Utilization of Chinese Herbal Medicine, Ministry of Education, Institute of Medicinal Plant Development, Chinese Academy of Medical Sciences and Peking Union Medical College, Beijing, China; ^2^Université de Lorraine, INRAE, UMR Interactions Arbres/Microorganismes, INRAE Grand Est - Nancy, Champenoux, France

**Keywords:** comparative transcriptome, *Tulasnella* sp., *Serendipita* sp., CAZymes, symbiotic germination

## Abstract

Orchid seed germination in nature is an extremely complex physiological and ecological process involving seed development and mutualistic interactions with a restricted range of compatible mycorrhizal fungi. The impact of the fungal species' partner on the orchids' transcriptomic and metabolic response is still unknown. In this study, we performed a comparative transcriptomic analysis between symbiotic and asymbiotic germination at three developmental stages based on two distinct fungi (*Tulasnella* sp. and *Serendipita* sp.) inoculated to the same host plant, *Dendrobium officinale*. Differentially expressed genes (DEGs) encoding important structural proteins of the host plant cell wall were identified, such as epidermis-specific secreted glycoprotein, proline-rich receptor-like protein, and leucine-rich repeat (LRR) extensin-like protein. These DEGs were significantly upregulated in the symbiotic germination stages and especially in the protocorm stage (stage 3) and seedling stage (stage 4). Differentially expressed carbohydrate-active enzymes (CAZymes) in symbiotic fungal mycelium were observed, they represented 66 out of the 266 and 99 out of the 270 CAZymes annotated in *Tulasnella* sp. and *Serendipita* sp., respectively. These genes were speculated to be involved in the reduction of plant immune response, successful colonization by fungi, or recognition of mycorrhizal fungi during symbiotic germination of orchid seed. Our study provides important data to further explore the molecular mechanism of symbiotic germination and orchid mycorrhiza and contribute to a better understanding of orchid seed biology.

## Introduction

On Earth, ~90% of angiosperm plants can form a mycorrhizal symbiosis with more than 50,000 fungal species belonging to Ascomycota, Basidiomycota, Glomeromycotina and Mucoromycotina (Bruns et al., 2018; Tedersoo et al., 2020). The most ubiquitous mycorrhizal type are arbuscular mycorrhizae (AM, 72%), orchid mycorrhizae (ORM, 10%), ectomycorrhizae (ECM, 2%), and ericoid mycorrhizal (ERM, 1.4%) (Genre et al., 2020). Similar to ancient AM, the orchid mycorrhiza also partakes in endosymbioses with specific intracellular structures, but orchid plants have lost the ability to form arbuscular mycorrhizal symbiosis and represent clear symbiosis switches that have occurred during plant evolution (Radhakrishnan et al., 2020). Orchid mycorrhizal fungi transfer carbohydrates to their hosts, especially during the early germination and seedling stages, whereas other types of mycorrhizal fungi (e.g., AM) primarily transport inorganic matter to host plants (Cameron et al., 2006; Parniske, 2008).

Because of their highly ornamental and medicinal value, orchid plants around the world are facing serious extinction so there is an urgent need to conserve them. The *Dendrobium* genus is one of the largest genera in the orchid family, and many species, such as *Dendrobium officinale*, are traditionally used in Chinese medicine. Orchid seeds generally lack endosperm and rely on mycorrhizal fungi to provide essential nutrients for germination, protocorm growth, and even adult plant development (Rasmussen et al., 2015). Most studies indicate that symbiotic germination is an effective technique for orchid conservation and ecological restoration in natural habitats (Phillips et al., 2020; Shao et al., 2021). In recent years, a large number of orchid mycobionts have been identified worldwide. These identifications, mainly based on molecular biology approaches such as high-throughput sequencing, have shown that the diversity and composition of orchid mycorrhizal fungi are related to plant species, geographical distribution, nutrition type, ecotype, and even developmental stage (Xing et al., 2015; Jacquemyn et al., 2017; Freestone et al., 2021). The most common orchid mycorrhizal fungi are saprotrophic basidiomycetes of the Ceratobasidiaceae, Tulasnellaceae, and Serendipitaceae families, which were previously assigned to asexual rhizoctonias (Rasmussen, 2002; Dearnaley, 2007; Smith and Read, 2008; Li et al., 2021). Yet, many mycoheterotrophic orchids have been reported to be associated with ectomycorrhizal fungi (Taylor and Bruns, 1997; Suetsugu et al., 2020). Some orchids are associated with root endophytes (Selosse et al., 2021). Although great progress has been made on the mycorrhizal diversity, specificity, and ecological dynamics of orchid-fungal association, orchids and their mycorrhizal partner are underexplored compared to the well-studied AM and ECM (Jacquemyn et al., 2015; McCormick et al., 2018; Li et al., 2021; Ventre Lespiaucq et al., 2021; Wang et al., 2021). Several important works have tried to elucidate the morphological and metabolic changes during the symbiotic process (Smith and Read, 2008; Ghirardo et al., 2020), nutrients exchange (Bougoure et al., 2010; Kuga et al., 2014; Dearnaley and Cameron, 2017; Suetsugu et al., 2020), genes or proteins expression (Balestrini et al., 2014; Perotto et al., 2014; Chen et al., 2017, 2020; Fochi et al., 2017; Adamo et al., 2020; Favre-Godal et al., 2020; Valadares et al., 2021) and common and unique trait of mycorrhizal symbiosis (Genre et al., 2020). Recently, the expression analysis of calcium and calmodulin-dependent protein kinase gene CCaMK, homologs of AM-related genes in orchids, suggested orchids possess, at least in part, the molecular mechanisms common to AM plants (Miura et al., 2018). However, the orchid mycorrhizal process was speculated to have unique characteristics due to the different taxonomic and genetic features of the partners. Thus, additional work is needed to fully understand orchid mycorrhizal biology.

The germination of orchid seeds is extremely complex as they are obligated to associate with mycorrhizal fungi for carbon source supply and are moreover influenced by abiotic factors. Although the fungal function for nutrition supply to the seed germination has been speculated and verified in early studies (Smith and Read, 2008), little information regarding the symbiotic molecular mechanism, especially in the establishment of symbiotic processes and functional pathways, is available. Before mycorrhizal fungi reach their full symbiotic functionality (to stimulate seed germination), the penetration and development of an extensive contact surface between the plant and fungal cells are essential, as this is where the transport and exchange of nutrients occur between plant cells and fungi take place (Balestrini and Bonfante, 2014; Rich et al., 2014). It has been demonstrated that high carbon transfer occurs in the interface between host roots and the AM fungal partner (Vandenkoornhuyse et al., 2007) and recently, a sucrose transporter has been documented to mediate sucrose import at the symbiotic interface for carbon allocation of heterotrophic *Gastrodia elata* with *Armillaria* (Ho-Pl Garo et al., 2021). Plant cell walls are thought to play a central role in mycorrhizal symbiosis. It was confirmed that the penetration of the fungus into the cortical cell of the orchid triggers the change of the plant's cytoskeleton (Uetake et al., 1996). The actin cytoskeleton also undergoes major modifications in cortical cells and the dynamic of the cytoskeleton in orchid mycorrhizal protocorms has been described in detail in early studies (Uetake and Peterson, 1998). Moreover, observations and immunolocalization by electron microscopy indicated that components such as xyloglucans, proteins rich in hydroxyproline (HRGPs), and arabinogalactan proteins (AGPs) are localized in the cell wall and interfacial matrix near the fungal cell wall during fungal colonization in the embryonic cells of orchid seeds (Li et al., 2018).

Moreover, increasing comparative genomics analysis of mycorrhizal and saprophytic fungi have attempted to create toolkits of mycorrhizal symbiosis from a fungal perspective. Large-scale genome sequencing of fungi of different lifestyles, including mycorrhizal fungi and saprotrophic, endophytic, and pathogenic species, has also demonstrated that the transition of fungal lifestyles from saprophyte to symbiosis involves widespread losses of lignin- and cellulose-acting degrading enzymes and the diversification of novel lineage-specific genes induced by symbiosis (Kohler et al., 2015; Miyauchi et al., 2020). However, orchid mycobionts in Tulasnellaceae, Serendipitaceae, and Ceratobasidiaceae possess large sets of carbohydrate-active enzymes (CAZymes) acting on cellulose, hemicellulose, and pectins that support their saprotrophic ability (transfer carbohydrates to their hosts), especially during the early stage of seed germination; thus, the nutritional strategies of orchid plant and the characteristics of their mycorrhizal fungi likely dictate to some extent the specificity for symbiotic association (Miyauchi et al., 2020).

During fungal colonization in orchid seeds or roots, fungal hyphae must penetrate into the cell walls of the epidermis or root hairs to enter cortical cells (Chen et al., 2014; Favre-Godal et al., 2020). Meanwhile, fungi release a wide range of extracellular enzymes to degrade plant cell walls (e.g., CAZy glycoside hydrolases, GH families) or secrete effector proteins to inhibit plant defense and help them achieve successful colonization (e.g., Small Secreted proteins, SSPs) (Pellegrin et al., 2015; Feldman et al., 2020; Tanaka and Kahmann, 2021). Furthermore, several SSPs have been identified as fungal effectors that play key roles in ECM, AM, and EM symbiosis (Plett et al., 2014; Casarrubia et al., 2018; Zeng et al., 2020). Recently, Adamo et al. (2020) attempted to elucidate the behavior of saprotrophic fungi in orchid mycorrhiza from expression changes of the fungal genes encoding degrading enzymes of the plant cell wall (PCW) between saprotrophic growth and mycorrhizal symbiosis of *Tulasnella calospora* and suggesting fungal PCW-degrading enzymes is finely regulated during saprotrophic growth and in symbiosis, often with a different regulation in the two orchid species. Thus, the role of the fungal PCW degrading enzymes in orchid symbiotic interactions are need to be addressed furtherly.

Thus, to further investigate the molecular responses of plants to mycorrhizal fungal colonization during the symbiotic germination of orchid seeds, we performed a comparative transcriptomic analysis between symbiotic and asymbiotic germination trials at three developmental stages based on two different fungi (*Tulasnella* sp. or *Serendipita* sp.), which were inoculated on the same host plant. We emphasize (i) plant gene expression changes related to plant cell wall biosynthesis, and structure modification, as well as (ii) the expression profile of fungal genes related to plant/fungal cell wall degradation (i.e., CAZymes). We aimed to (1) understand the molecular responses at the transcriptional level of *D. officinale* seeds after inoculation with two different fungal species and identify the core plant and fungal gene sets involved in symbiotic germination and (2) analyze the possible role of fungal genes encoding CAZymes which are known to be important for AM and ECM mycorrhizal establishment in orchid mycorrhizae.

## Materials and Methods

### Seed Sample Collection

Mature and indehiscent *D. officinale* capsules were collected from an artificial cultivation greenhouse in Jinhua, Zhejiang Province, in November 2018. The obtained capsules were dried naturally at room temperature (25°C) for ~1 week. Next, seeds were cleaned from capsule debris, mixed, and stored in wax paper on silica gel at 4°C. Two mycorrhizal fungi, *Tulasnella* sp. (strain no. S6) and *Serendipita* sp. (strain no. 12825), were isolated from the roots of *Dendrobium* spp. and deposited in the Institute of Medicinal Plant Development, Chinese Academy of Medical Science. Free-living mycelium was cultivated on a PDA medium at 25°C in the dark.

### Seed Germination Experiments

**Figure 1:**
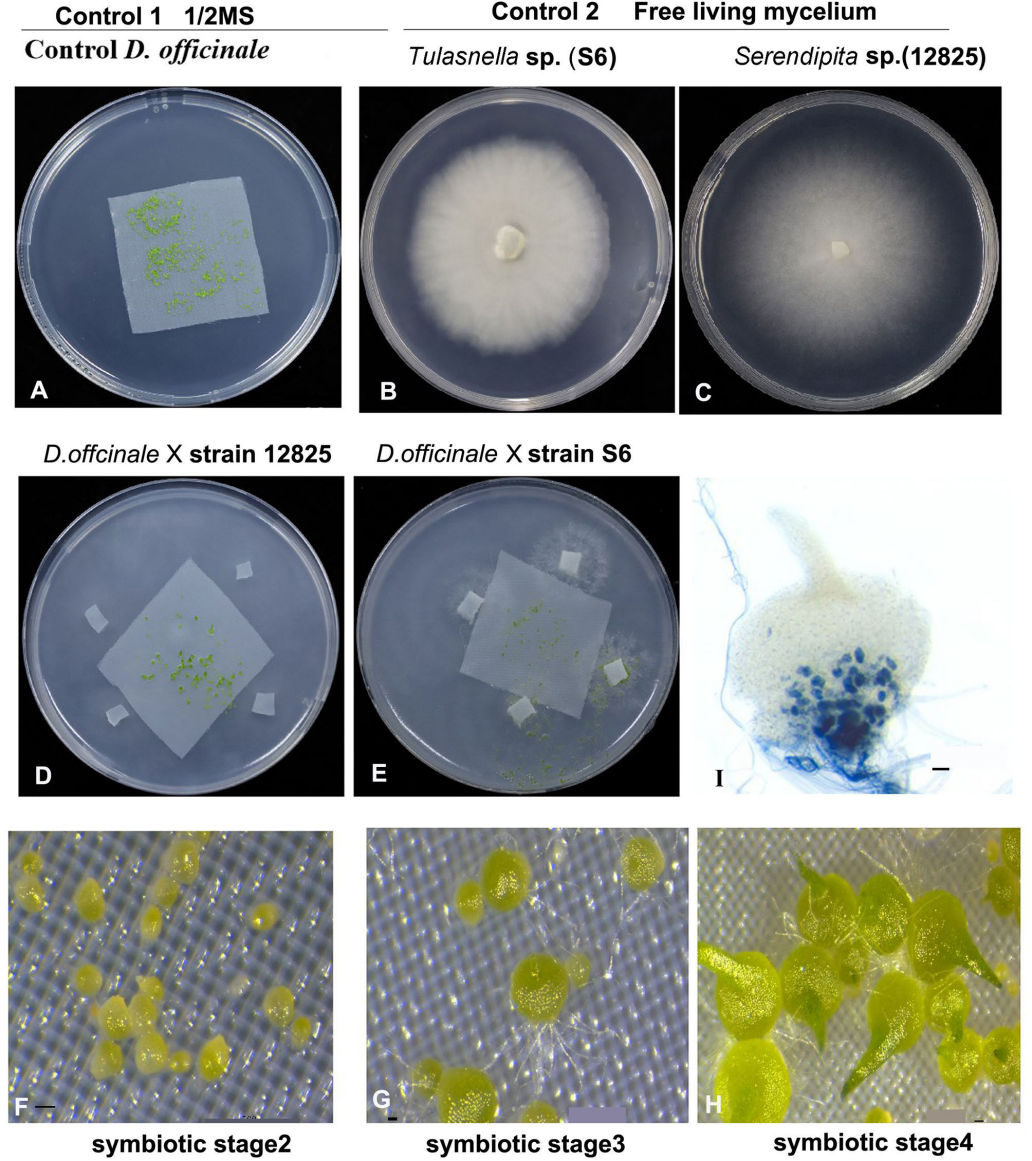
Experiment designs for our study. Aymbiotic and symbiotic germination of medicinal orchid *Dendrobium officinale* inoculated with two mycorrhizal fungi *Tulasnella* sp. (S6) and *Serendipita* sp. (12825) at three development stages. **(A)** showing asymbiotic germination in 1/2 MS medium as control; **(B,C)** The free-living mycelium of two fungi on PDA medium, respectively. **(D–H)** showing the symbiotic germination assay with two fungal species, respectively. **(I)** Trypan blue staining showing the pelotons in embryo cell in symbiotic stage 4 of *D. officinale* seed inoculated with strain 12825. Scale bar = 20 μm in **(F–H)**, and 50 μm in **(I)**.

The seed germination experiment was designed as shown in Figure 1. Before sowing, the seeds were immersed in sterile water and allowed to stand for 1–2 h. Seeds in the bottom of the tube were chosen for subsequent experiments. First, the seeds were surface sterilized in 1% NaClO for 3 min, rinsed three times, and diluted with sterile water into a suitable seed suspension for sowing. For symbiotic germination, 400 μL of seed suspension was dispensed with a pipette onto a square of autoclaved nylon cloth (4 × 4 cm) on oatmeal agar plates (OMA, 0.25% oatmeal, and 1% agar, 20 ml in total) in a 9 cm petri dish, and then four 5 × 5 mm inocula of *Tulasnella* sp. S6 (or *Serendipita* sp. 12825) on PDA medium were placed in each OMA for coculture. For asymbiotic germination, only 400 μL of seed suspension was dropped onto the 1/2 MS culture medium. Plates were incubated at 25°C with a 12 h/12 h light-dark cycle. Seed germination and protocorm development were evaluated under a dissecting stereomicroscope every 2 days. The seed developmental stages were defined according to the previous study by Stewart and Zettler (2002).

### Total RNA Extraction, Library Preparation, and Sequencing

Symbiotic (with *Tulasnella* sp. or *Serendipita* sp.), asymbiotic germination seeds at stage 2 (germination), stage 3 (protocorm), stage 4 (seedling), and free-living mycelium of both fungi were collected either immediately frozen in liquid nitrogen or stored at −80°C for RNA extraction. There are three biological replicates for each sample. Total RNA of 11 samples including three samples for asymbiotic, three symbiotic samples with *Tulasnella*, three symbiotic samples with *Serendipita* (stages 2, 3, and 4, respectively), and two free-living mycelium fungal samples *Tulasnella* sp. and *Serendipita* sp., respectively) was extracted using the RNeasy Plant Mini Kit (QIAGEN, Hilden, Germany), and the quality, integrity, and quantification of RNA were assessed following our previous study (Chen et al., 2017). Finally, 2 μg of high-quality total RNA (RNA Quality number, RON > 7) was used for stranded RNA sequencing library preparation. The library was constructed using a KC-Digital TM Stranded mRNA-seq Library Prep Kit for Illumina® (Kangce Technology Co., LTD, Wuhan, China) following the manufacturer's instructions. Duplication bias in PCR and sequencing steps were eliminated using a unique molecular identifier (UMI) of 8 random bases to label the preamplified cDNA molecules with this kit. The library products corresponding to 200–500 bps were enriched, quantified, and finally sequenced on an Illumina HiSeq 2500 sequencer (Illumina, San Diego CA, USA) using the paired-end (PE) 150 strategy (Kangce Technology Co., LTD, Wuhan, China).

Prior to assembly and mapping, the raw sequencing data were first filtered by Trimmomatic (version.36) (Bolger et al., 2014), and then the low-quality reads and the reads containing adapter were trimmed using default parameters (PE -phred33 ILLUMINACLIP:adaptor_file:2:30:5 LEADING:3 TRAILING:3 SLIDINGWINDOW:4:15 HEADCROP:0 MINLEN:36). Clean reads were further treated with in-house scripts to eliminate duplication bias introduced in library preparation and sequencing. Briefly, clean reads with the same UMI sequence were first clustered together, and then they were compared to each other using pairwise alignment. Reads with sequence identity over 95% were extracted into a new subcluster. After all subclusters had been generated, multiple sequence alignment was performed to obtain one consensus sequence for each subcluster. Using these steps, any errors and biases introduced by PCR amplification or sequencing were eliminated. The de-duplicated consensus sequences with high quality were used for downstream analyses.

### Analysis of *D. officinale* Transcriptomic Data

Genomics mapping and alignment program analysis were performed based on the reference genome of *Dendrobium catenatum* (https://www.ncbi.nlm.nih.gov/assembly/GCF_001605985.2) using STAR software (version 2.5.3a) with default parameters (Dobin et al., 2013; Dobin and Gingeras, 2015). Functional annotation of predicted genes was first performed using diamond Blastx in UniProt (Universal Protein) to obtain the protein ID, and then the annotation was searched in the database of Nr (NCBI non-redundant protein sequences), Pfam (Protein family), Rfam (RNA family), eggNog (evolutionary genealogy of genes: non-supervised orthologous groups), Gene Ontology (GO), and Kyoto Encyclopedia of Genes and Genomes (KEGG) using the same ID. Gene expression was qualified using the RSEM software package (RNA-Seq by Expectation-Maximization). Genes that were differentially expressed between groups were identified using the edgeR package (version 3.12.1) (Li and Dewey, 2011). The threshold for significantly differentially expressed genes (Robinson et al., 2010) (DEGs) was set at a fold change of ≥2 and an FDR corrected *p*-value < 0.05 (Robinson and Oshlack, 2010).

### Analysis of Orchid Mycorrhizal Fungi *Tulasnella* sp. and *Serendipita* sp. Transcriptomic Data

Fungal *de novo* transcriptome assemblies were first reconstructed using Trinity based on the free-living mycelium of *Tulasnella* sp. (S6) and *Serendipita* sp. (12825), and the longest transcript was chosen as the unigene. Next, the unmapped reads of symbiotic samples (after mapping to the plant genome) were subjected to mapping analysis using the unigenes by Kangce Technology Co., Ltd., Wuhan, China, followed by Grabherr et al. (2011). Because we focused our analysis on the gene scale, further analysis was performed on Trinity unigenes, i.e., the longest isoform per Trinity gene. TransDecoder (version 5.5; http://transdecoder.sourceforge.net/) was used to identify putative coding regions from the assembled unigenes, and only the best-predicted protein was retained for each transcript (single_best_only option). Carbohydrate active enzyme (CAZyme) assignment was performed with dbCAN2 (Zhang et al., 2018) using HMMER, DIAMOND, and Hotpep prediction. Only domains detected by at least two tools were conserved.

## Results

### Global Analysis of the Transcriptomic Data and Identification of Core Plant Gene Expression

**Figure 2:**
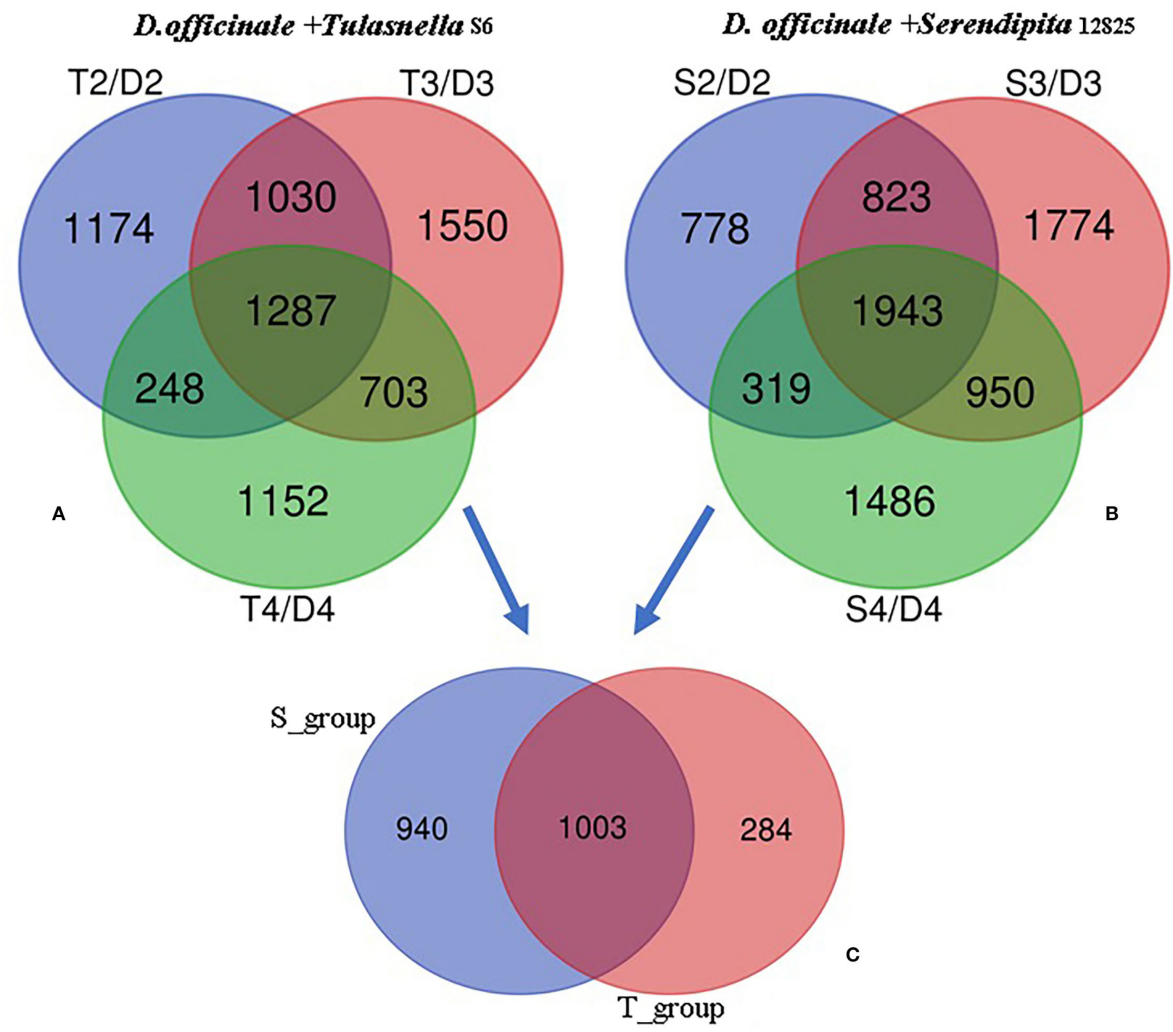
Venn diagram showing the numbers of plant differentially expressed genes (DEGs) in the adjacent development stage of two symbiotic groups of *D. officinale* seeds with *Tulasnella* sp. (S6) and *Serendipita* sp. (12825), respectively. **(A,B)** DEGs at adjacent development stage during *D.officinale* seed with *Tulasnella* sp. **(A)** and *Serendipita* sp. **(B)** and compared to the same stage in asymbiotic germination; **(C)** 1,003 common differentially expressed plant genes across the three development stages (stages 2, 3, and 4) in symbiotic process with two different mycorrhizal fungi compared asymbiotic germination. D2, D3, and D4 mean asymbiotic germination stages 2, 3, and 4, respectively; S2, S3, and S4 mean stages 2, 3, and 4 in symbiotic germination with *Serendipita* sp., respectively, T2, T3, and T4 mean stages 2, 3, and 4 in symbiotic germination with *Tulasnella* sp., respectively.

**Figure 3:**
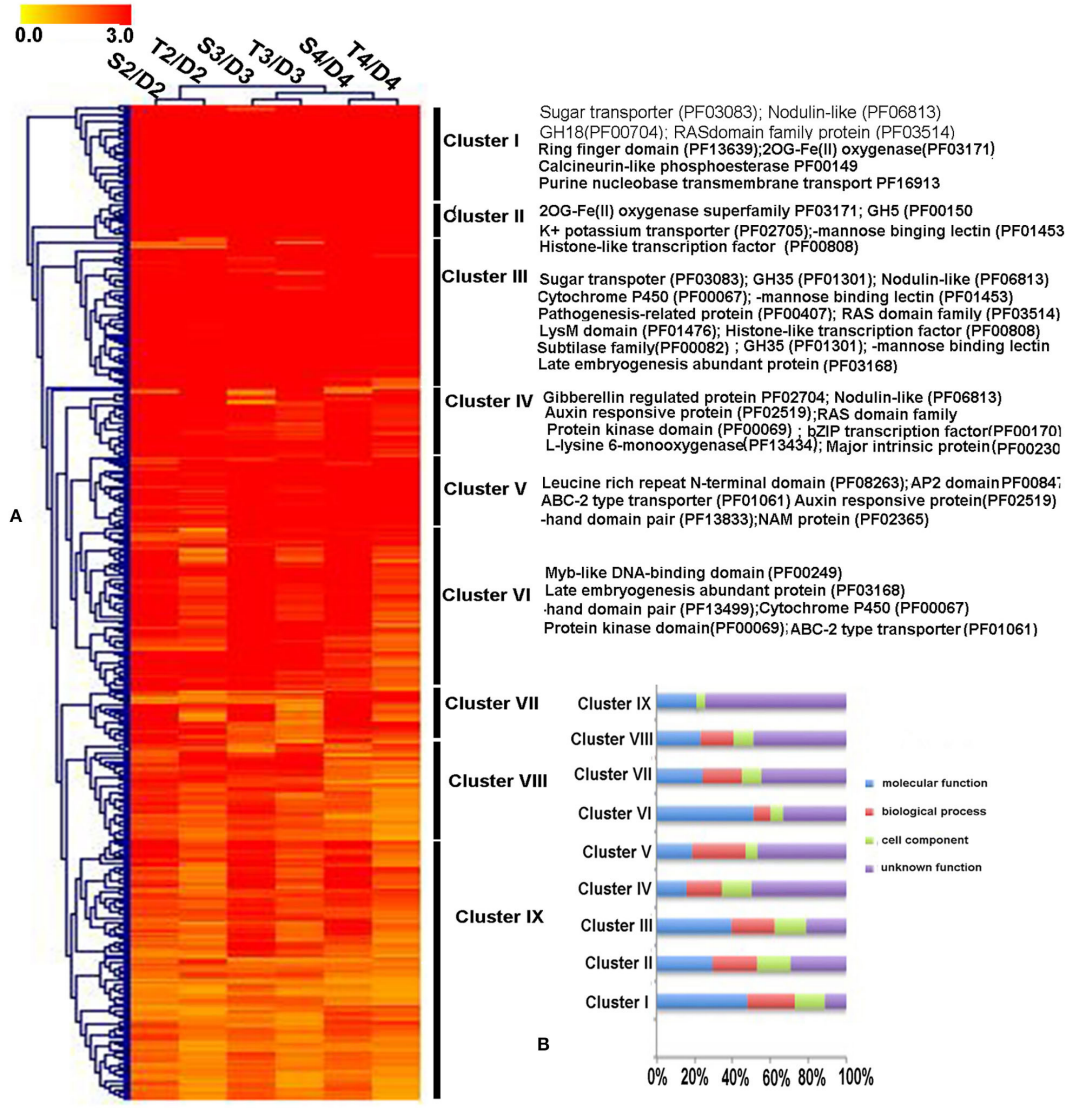
The expression patterns and functional classification of 452 differentially upregulated expressed plant genes (DEGs) at least one symbiotic stage during *D. officinale* inoculated two mycorrhizal fungi. **(A)** Heatmap showing the expression level of up-regulated genes in symbiotic germination of *D. officinale*. Expression value was calculated based on Log_2_ Fold change. Red color means a gene with a high expression value (Fold change ≥ 8) and yellow color means a gene with a relatively low expression value (Fold change ≥ 2). The function annotation of the representative genes with high expression (Cluster I-Cluster VI) was marked in the heatmap right. **(B)** GO functional category of the 452 up-regulated genes in nine clusters. D2, D3, and D4 mean asymbiotic germination stage2, stage3, and stage4, respectively; S2, S3, and S4 mean stages 2, 3, and 4 in symbiotic germination with *Serendipita* sp., respectively, T2, T3, and T4 means stages 2, 3, and 4 in symbiotic germination with *Tulasnella* sp., respectively. The detailed information of these genes is in Supplementary Table S1.

Transcriptomic data were generated for the symbiotic germination of *D. officinale* seeds with the two mycorrhizal fungal species, asymbiotic germination seeds, and free-living mycelium of both fungi (Figure 1; Table 1). An average of 64.52% of the total 24,811 plant genes were detected to have transcriptional activity in at least one of the symbiotic germination stages (RPKM value ≥ 5) of *D. officinale* seeds inoculated with *Tulasnella* sp. A total of 1,287 genes were common differentially expressed (|fold change| ≥ 2; FDR <.05) within the three symbiotic germination stages compared to that of asymbiotic germination (Figure 2A). Similarly, an average of 66.6% of plant genes exhibited expression values (RPKM value ≥ 5) in at least one of the symbiotic samples of *D. officinale* seeds inoculated with *Serendipita* sp., and 1,943 plant genes were commonly differentially expressed in the three symbiotic stages compared to those of asymbiotic germination (Figure 2B). When we examined gene expression between both symbiotic germination groups of *D. officinale* inoculated with *Tulasnella* sp. and *Serendipita* sp., respectively, 1,003 common regulated plant genes were identified (Figure 2C). Among 1,003 differentially expressed genes (DEGs), 452 genes were upregulated in symbiotic groups and were clustered into nine clades based on expression profile (Figure 3; Supplementary Table S1). The genes with the highest expression level were primarily restricted to clusters I-V (Figure 3), and they displayed highly similar expression profiles in symbiotic plants inoculated with the two different mycorrhizal fungi, including the genes encoding a nodulin-like protein, Ras domain protein, sugar transporter, lysM domain protein, mannose-binding lectin, and histone-like transcription factor. These upregulated genes took part in molecular functions, biological processes, and cell components.

**Table 1:** Samples information in our study.

**Development stage of *D. officinale* seeds**	**Sample label**				
	**Asymbiotic**	**Symbiotic**			
		**+*****Tulasnella*** **sp. (S6)**		**+*****Serendipita*** **sp. (12825)**	
	**For plant or fungus (control)**	**For plant**	**For fungus**	**For plant**	**For fungus**
Stage2 (germination)	D2	T2	Ft2	S2	Fs2
Stage3 (protocorm)	D3	T3	Ft3	S3	Fs3
Stage4 (seedling)	D4	T4	Ft4	S4	Fs4
Free-living mycelium for *Tulasnella* sp. (S6)	Ft				
Free-living mycelium for *Serendipita* sp. (12825)	Fs				

**Figure 4:**
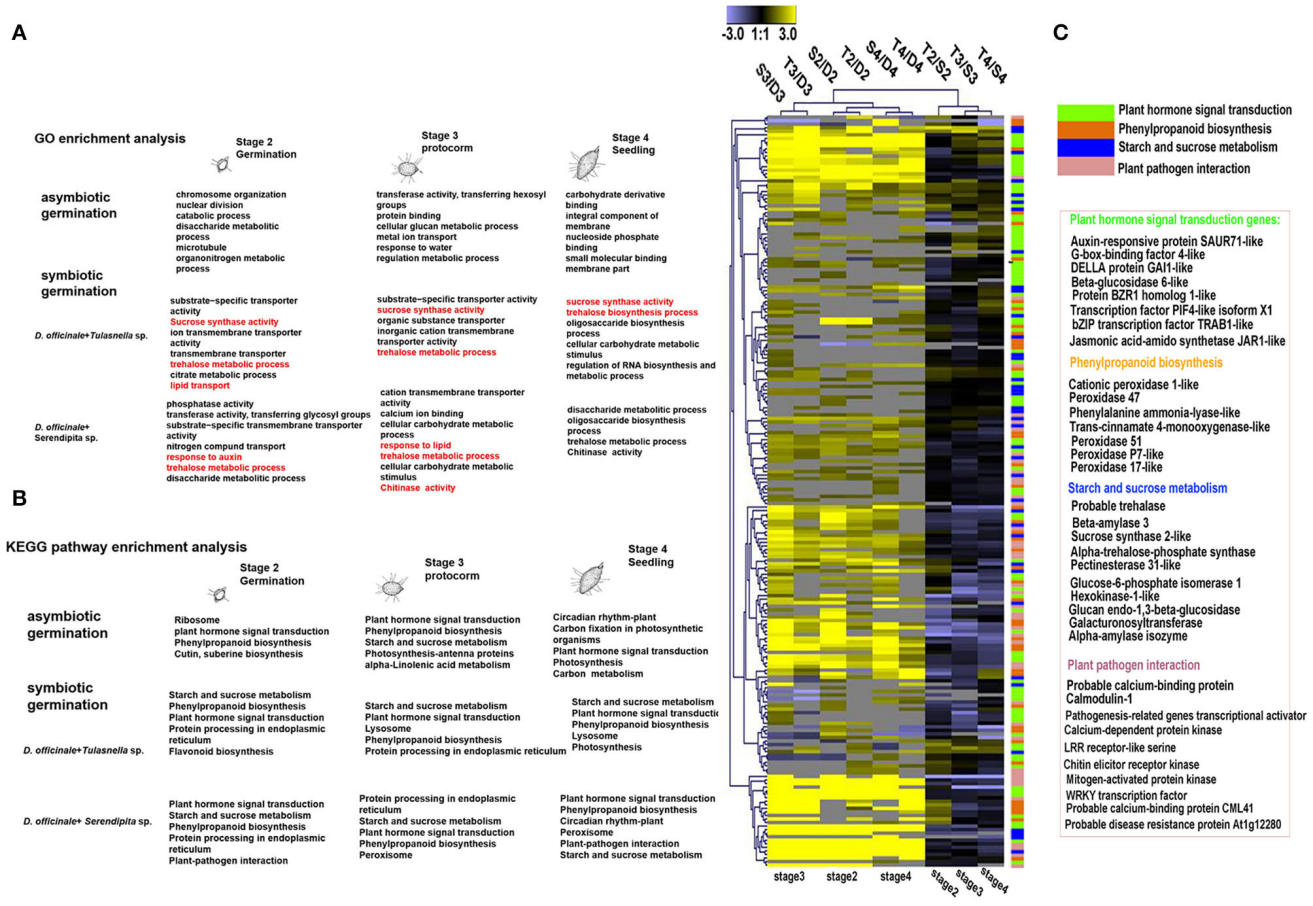
KEGG and GO enrichment analysis of differentially expressed plant genes (DEGs) during germination of *D. officinale* seeds with two mycorrhizal fungi. **(A)** GO enrichment analysis; red color indicated that typical Go functional enrichment for these genes involved in symbiotic germination compared to asymbiotic germination. **(B)** KEGG pathway enrichment analysis showed that the significant enrichment pathway exists as overlaps between symbiotic and asymbiotic germination. It means these pathways could be most important during seed development. And the symbiotic germination with two fungal species have similar metabolic pathway but also display difference e.g., lysosome in *Tulasnella* sp.-*D.officinale* and peroxisome in *Serendipita* sp*.- D.officinale*; **(C)** heatmap showing the expression profile of genes in the significantly enriched four metabolic pathways during symbiotic germination of *D.officinale* (such as plant hormone signal transduction, phenylpropanoid biosynthesis, starch, and sucrose metabolism and plant-pathogen interaction); expression value was calculated based on Log2 Fold change. Significant expression changes were labeled in colors: blue represents down regulated and yellow represents up regulated. The functional annotation of representative genes in each enriched pathway was recorded in heatmap aside.

To further explore commonly expressed plant genes of *D. officinale* seeds induced by both fungal species, GO function and KEGG pathway enrichment analyses were performed (Figure 4). Compared to asymbiotic germination, all DEGs across various developmental stages of symbiotic *D. officinale* seeds were primarily enriched in organic or inorganic substance transport and sucrose, trehalose, and lipid synthesis and transport (Figure 4A). Several pathways, such as plant–pathogen interactions, plant hormone signal transduction, starch, and sucrose metabolism and phenylpropanoid biosynthesis, protein processing in the endoplasmic reticulum, lysosomes (for *Tulasnella* sp. pairs), and peroxisome (for *Serendipita* sp. pairs) were significantly enriched, and genes involved in these pathways exhibited a similar pattern in interaction with both *Tulasnella* sp. and *Serendipita* sp. (Figures 4B,C). Most genes in the plant-pathogen interaction pathway were encoded by calcium-binding proteins, calmodulin, LRR receptor-like, WRKY transcription factor, etc. The genes encoding auxin-responsive proteins and DELLA proteins (involved in plant hormone signal transduction) were significantly enriched during the interaction between *D. officinale* seeds and their mycelia. Genes encoding trehalase, beta-amylase, pectinesterase, hexokinase, glucan endo-1,3-beta-glucosidase, etc., which are implicated in starch and sucrose metabolism, were also enriched in symbiotic germination from early germination to seedling formation. Twenty-five upregulated common expression genes of the host plant (fold change ≥ 5, FDR ≤ 0.05, compared to asymbiotic germination) are listed in Supplementary Table S2, and these genes typically encoded aquaporin-like, cytochrome P450, nodulin-like, glycoside hydrolases 35 (GH35), GH18, sugar transporter, etc.

**Figure 5:**
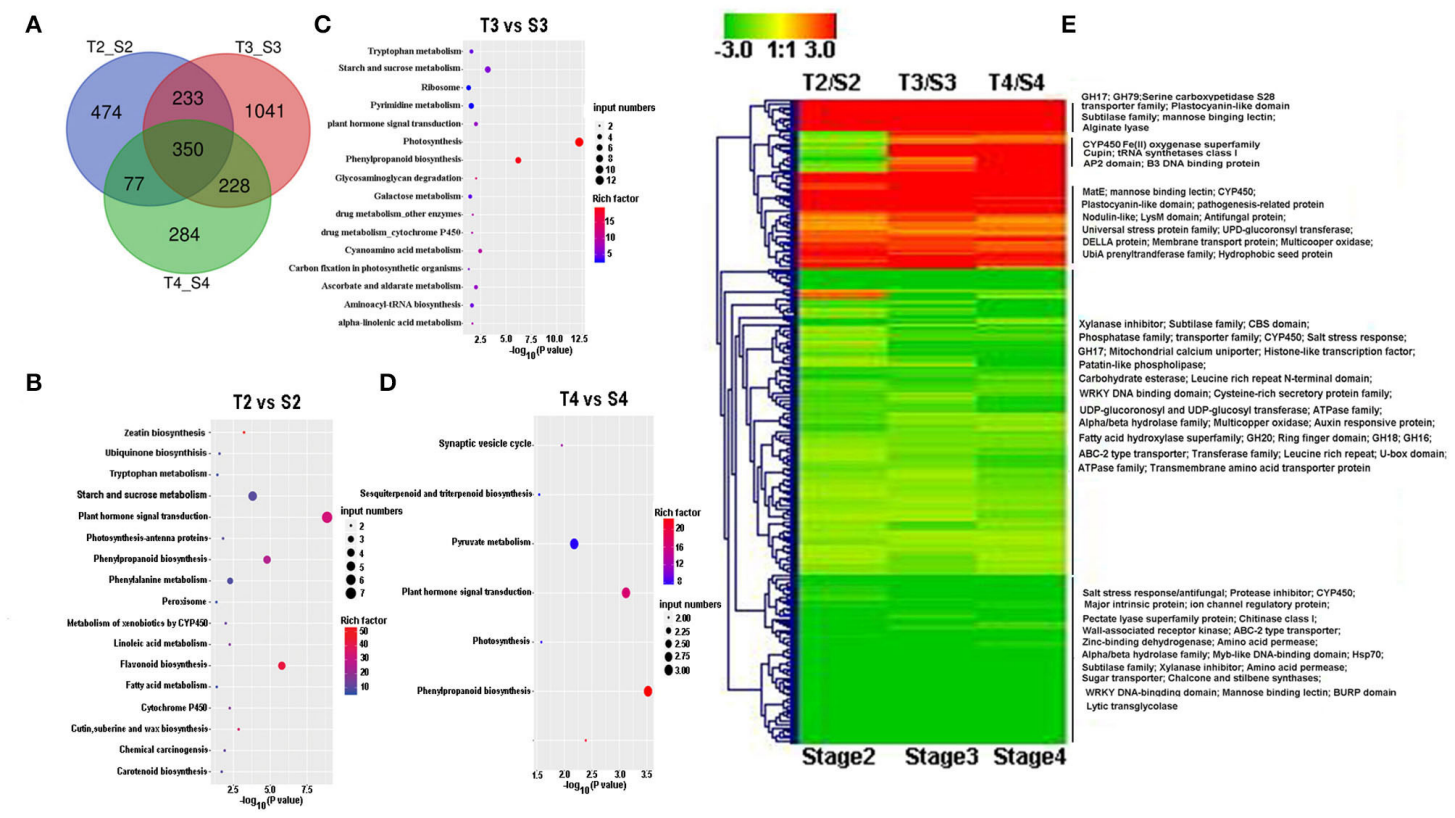
Heatmp showing the differentially expressed genes (DEGs) between symbiotic germination at the same stage during *D. offcinale* inoculated with *Tulasnella* sp. (S6) and *Serendipita* sp. (12825). **(A)** The number of DEGs when *D. officinale* seed inoculated with *Tulasnella* sp. compared to with *Serendipita* sp. at the same development stage; **(B–D)** KEGG enrichment analysis of up-regulated DEGs at various germination stages during *D.officinale* symbiotic germination with two fungi. **(E)** Heatmap showing the expression pattern of 350 common differentially expressed genes in *D. officinale* × *Tulasnella* sp. compared to *D. officinale* × *Serendipita* sp. Expression value was calculated based on Log2 Fold change, red color means up-regulated expression and green color means down-regulated expression. S2, S3, and S4 mean stages 2, 3, and 4 in symbiotic germination with *Serendipita* sp., respectively, T2, T3, and T4 mean stages 2, 3, and 4 in symbiotic germination with *Tulasnella* sp., respectively.

When we compared gene expression at the same germination stage of *D. officinale* seeds between inoculation with *Tulasnella* sp. and *Serendipita* sp., we found more DEGs in the symbiotic protocorm stage (1,852 genes, stage 3) than in the germination stage (934 genes, stage 2), implicating the protocorm stage is likely a crucial stage responding to different fungal invasions during orchid mycorrhizal development. In total, 350 plant genes were identified as significantly differentially expressed in symbiotic seeds of *D. officinale* across all germination stages during interaction with *Tulasnella* sp. compared to those symbiotic with *Serendipita* sp. (Figure 5). GH17, GH79, UPD-glucoronsyl transferase, UbiA prenyltransferase, CYP450, nodulin-like, hydrophobic seed protein, etc., were significantly upregulated in *D. officinale* seeds inoculated with *Tulasnella*, and upregulated plant genes in various symbiotic stages inoculated with *Tulasnella* sp. were primarily enriched in plant hormone signal transduction and phenylpropanoid biosynthesis, especially during the initial germination stage and photosynthesis pathway, and were significantly enriched in the protocorm stage (Figure 5).

### Genes Expression Involved in Plant Cell Wall Synthesis and Remodeling

We identified 15 common DEGs involved in plant cell wall constitution and remodeling at each symbiotic stages (Table 2) during seed germination of *D. officinale* inoculated with fungi. Among them, genes encoding epidermis-specific secreted glycoprotein, proline-rich receptor-like protein, and leucine-rich repeat (LRR) extensin-like protein were upregulated in the symbiotic stages and even more highly upregulated in the protocorm stage (stage 3) and seedling stage (stage 4). The gene encoding LRR extensin-like protein (LOC110096373) exhibited the highest expression of 20.2-fold in the protocorm stage of *D. officinale* seeds inoculated with *Serendipita* sp., and the gene encoding proline-rich receptor-like protein kinase displayed more highly upregulated expression with more than a 25-fold induction in the symbiotic stage compared to asymbiotic germination inoculated with either *Tulasnella* or *Serendipita* across the entire germination stage. Moreover, genes encoding enzymes that function in cell wall biosynthesis also displayed significantly differential expression between symbiotic and asymbiotic germination, such as cellulose syntheses, pectinacetylesterase, and pectinesterase. The gene encoding microtubule-associated protein RP was also significantly upregulated during symbiotic germination.

**Table 2:** Fifteen differentially expression plant genes involved in cell wall composition and remodeling during *D. officinale* symbiotic germination.

**Geneid**	**logT2/D2**	**logT3/D3**	**logT4/D4**	**logS2/D2**	**logS3/D3**	**logS4/D4**	**Description**
LOC110109313	4.31	5.00	3.24	4.60	6.05	4.13	Cellulose synthase-like protein D2
LOC110106368	−1.52	−3.72	−1.46	−3.27	−2.60	−1.75	Cellulose synthase-like protein G3
LOC110106369	−2.02	−2.00	1.21	−2.78	−1.83	1.40	Cellulose synthase-like protein G3
LOC110106370	−2.33	−2.18	−1.34	−3.44	−2.43	−1.94	Cellulose synthase-like protein G3
LOC110096247	1.15	1.63	1.09	1.28	2.30	1.62	Microtubule-associated protein RP
LOC110113646	−1.87	−2.84	−1.62	−2.23	−2.23	−1.12	Pectinesterase inhibitor 11-like
LOC110101432	−1.90	−3.42	−2.25	−2.21	−3.72	−3.19	Pectinesterase inhibitor 3-like
LOC110112046	1.27	1.05	2.38	1.53	1.03	2.20	Pectinesterase-like
LOC110101606	3.69	4.99	3.30	4.48	5.98	4.61	Probable pectinesterase
LOC110115556	2.05	2.47	2.40	2.44	1.47	1.67	Epidermis-specific secreted glycoprotein
LOC110113177	−1.18	−1.86	−1.70	−2.78	−3.17	−1.88	36.4 kDa proline-rich protein-like
LOC110094976	4.65	4.90	4.49	5.12	5.99	6.08	Proline-rich receptor-like protein kinase PERK1
LOC110093563	−1.03	−2.03	−1.95	−1.52	−1.04	−1.15	Extensin-2-like
LOC110103208	−2.78	−2.25	−2.24	−3.87	−1.22	−2.33	Extensin-3-like isoform X1
LOC110096373	2.89	2.65	2.12	3.51	4.34	3.45	Leucine-rich repeat extensin-like protein 5

### Fungal Gene Expression During Interaction With the Same Host Plant

#### Overview of Fungal Genes Expression During Seed Symbiotic Germination of *D. officinale*

With the seed germination process occurring in the symbiotic system, fungal mycelium in embryonic cells also underwent a series of morphological changes. Although the mycorrhizal infection in orchid protocorm could be a cyclic event and pelotons are supposed as short-lived structures (Smith and Read, 2008), our previous showed that in the early stage of seed germination (embryo enlargement, rupture of testa, stage 2), several fungal mycelia colonized the embryonic cells from the epidermal hair or suspensor, and the hyphae formed pelotons with protocorm development (stage 3). After protocorm formation, most invaded hyphae lost bioactivity and formed clumps that started to degenerate at stage 4 or 5 (seedling development), although the timepoint boundary was not entirely clear (Chen et al., 2014).

To analyze fungal gene expression, RNA-seq data were generated from fungi in symbiotic status, namely, colonized during three symbiotic germination stage samples (seed germination, stage 2; protocorm formation, stage 3, and seedling development stage 4) and compared to free-living mycelium (FLM). Assembly of the fungal transcriptome was based on our *de novo* sequencing of *Tulasnella* sp. (S6) and *Serendipita* sp. (12825) due to the low species similarity with the published genome, but gene annotation was also performed with the reference genome of *Tulasnella calospora* AL13/4D (version 1; https://www.ncbi.nlm.nih.gov/assembly/GCA_000827465.1/) and *Serendipita vermifera* MAFF 305830 (version 1; https://www.ncbi.nlm.nih.gov/assembly/GCA_000827415.1/).

In the global expression analysis of *Tulasnella* sp. during interaction with *D. officinale* seeds, a total of 8,228, 7,939, and 6,034 genes exhibited transcriptional activities at the beginning of invasion (according to stage 2 of seed germination), peloton formation (stage 3), and peloton degradation stages (stage 4), respectively. Among them, 2,555 common upregulated genes were identified across the entire symbiotic process with *D. officinale* seeds (compared to FLM) (Supplementary Figure S1). Similarly, there were 4,630, 4,182, and 3,123 genes differentially expressed in symbiotic stages 2, 3, and 4, respectively, compared to FLM when *D. officinale* seeds were inoculated with *Serendipita* sp. (Supplementary Figure S1). Furthermore, 403 fungal genes were commonly upregulated in the symbiotic process across the three seed germination stages compared to the FLM of *Serendipita* sp. (Supplementary Figure S1). The number of DEGs in *Serendipita* sp. (403 common upregulated) was less than that of *Tulasnella* sp. (2,555 common upregulated genes). Functional analysis of the two upregulated fungal gene sets revealed that the most abundant genes with known functions played important roles in transcription, posttranslational modification, lipid transporter, and metabolism, carbohydrate transport and metabolism, amino acid transport, and metabolism, and RNA processing and modification (Supplementary Figure S1).

### Expression Profile of Fungal Genes Encoding Carbohydrate- Active Enzymes

**Figure 6:**
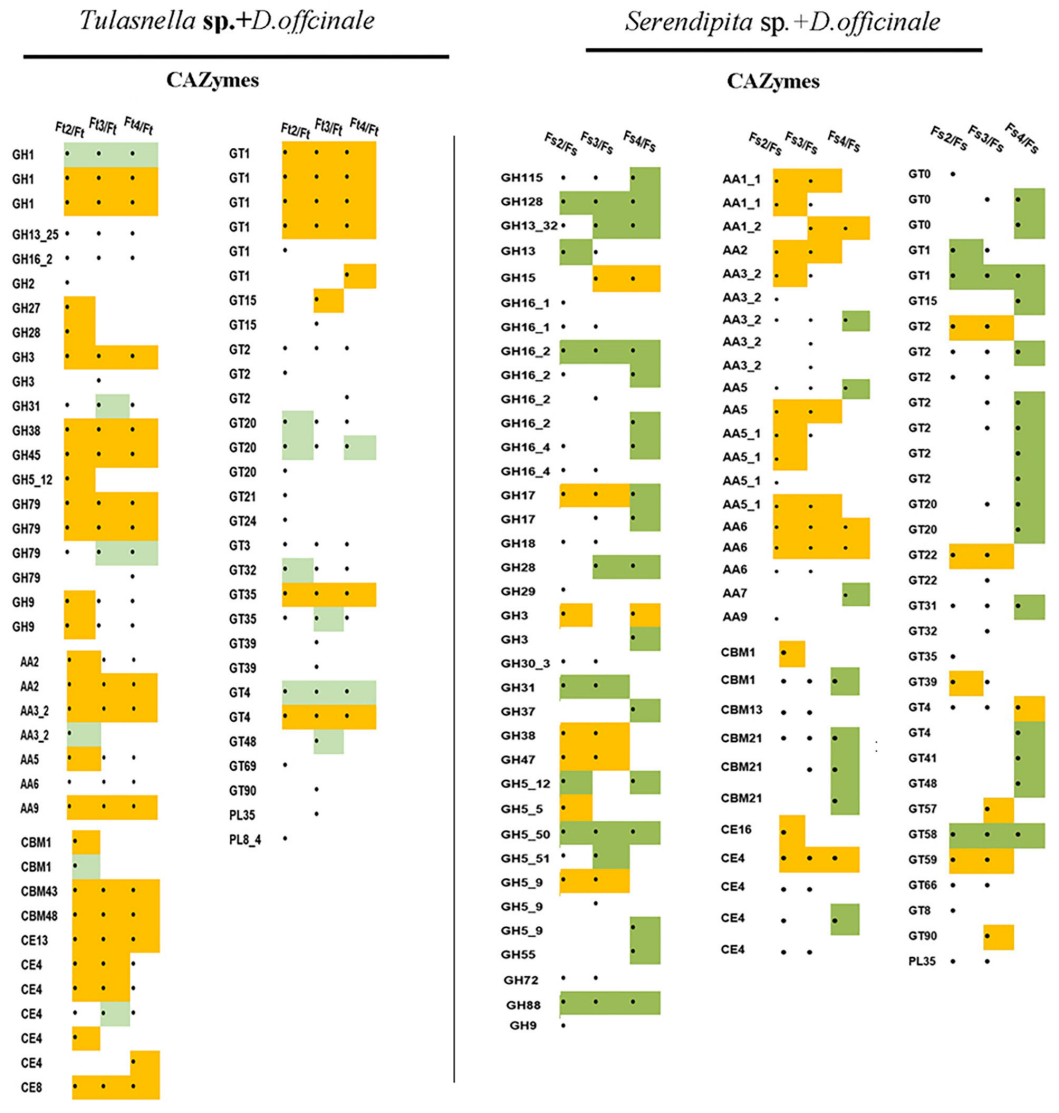
Heatmap showing the expression profile of fungal DEGs encoding CAZyme proteins during symbiotic germination of *D.officinale* with *Tulasnella* sp. and *Serendipita* sp. ∙ means genes differentially expression, blank means no significant differential expression; yellow color means genes up-regulated with fold change ≥ 5 and green color means genes down regulated with fold change ≥ 5. Ft2, Ft3, and Ft4 represent *Tulasnella* sp. in symbiotic statuses 2, 3, 4 stages while Fs2, Fs3, and Fs4 represent *Serendipita* sp. in symbiotic statuses 2, 3, and 4 stages (according to germination stage) and Ft and Fs means free-living mycelium of *Tulasnella* sp. and *Serendipita* sp., respectively.

Based on our transcriptomic data, we identified 266 putative genes encoding CAZymes in the *Tulasnella* sp. × *D. officinale* symbiotic transcriptome, and among them, 66 genes were differentially expressed in at least one symbiotic stage compared to that of the FLM (Figure 6), including 3 carbohydrate-binding modules (CBM) family members (4 genes), 3 members of the carbohydrate esterase (CE) family (7 genes), 5 members of the auxiliary activity (AA) (7 genes), 13 glycoside hydrolase (GH) family members (20 genes), 14 glycosyltransferases (GT) family members (27 genes), and two pectin lyase members (PL35, PL8). Moreover, CBM43, CBM48, CBM13, CE8, AA2, AA9, GH1, GH79, GH38, and GH45 were upregulated in the entire symbiotic stage with more than 5-fold changes compared to that of the FLM.

Compared to the interaction between *Tulasnella* sp. × *D. officinale* seeds, the transcriptomics analysis of *Serendipita* sp. × *D. officinale* identified 270 putative genes encoding CAZymes. Among them, 99 genes were differentially expressed in the symbiotic stage (Figure 6). Genes encoding AA class proteins (21 genes for 7 members) targeted to pectin were significantly upregulated in symbiotic conditions by more than 5-fold compared to free living mycelium. In addition, genes in the GH family, such as GH15, GH17, GH3, GH5, GH38, GH47, and GT family members (GT2, GT22, and GT59), were upregulated in the symbiotic stage compared to FLM.

### Definition of a Core Gene Set Shared by *Tulasnella* sp. and *Serendipita* sp. During Interaction With the Same Host Plant

We hypothesized that the different mycorrhizal fungal species would share common gene expression patterns during interaction with seeds of the same host plant of *D. officinale*. To identify possible orthologous genes between the two mycorrhizal fungi, we performed a bidirectional BlastP analysis on *Tulasnella* sp. and *Serendipita* sp. at an e-value < 10^−5^. Additionally, we performed homology analysis using BlastP search with the published *Tulasnella* (AL13/4D version 1) and *Serendipita* (MAFF 305830 v1.0) genomes for gene annotation.

We identified 4,954 genes (25.21%) that were orthologous between *Tulasnella* sp. (S6) and *Serendipita* sp. (12825) based on the fungal transcriptomics data. Among them, 1,682 genes were expressed in symbiotic *Serendipita* sp. and 2,464 genes were expressed in symbiotic *Tulasnella* sp. in at least one symbiotic stage during the interaction with *D. officinale* seeds. A total of 936 orthologous genes were shared between *Tulasnella* sp. (S6) and *Serendipita* sp. (12825) in at least one symbiotic stage during colonization in seeds of *D. officinale*. Most of these genes exhibited similar expression profiles. According to gene expression level, 936 orthologous genes were divided into five clusters (Supplementary Figure S2; Supplementary Tables S2, S3). For example, in cluster 1, genes were primarily upregulated in both fungi during early symbiotic stages with seeds (stage 2, starting germination), and most of these genes were involved in energy production and conservation, translation and posttranslational modification, amino acid transport, and metabolism and lipid transport and metabolism. Genes encoding a fungal transcriptional regulatory protein, chitin synthase, zinc finger protein, serine/threonine-protein kinase-related, ribosomal protein, transcription factor, sugar transporter, ABC transporter-like, and acyltransferase, which participate in signal transduction, posttranslational modification, lipid transport and metabolism (androgen and estrogen metabolism), were upregulated in symbiotic *Tulasnella* sp. but downregulated in symbiotic *Serendipita* sp. during the entire process of interaction with *D. officinale* seeds (cluster 2), especially during the germination and protocorm stages (stages 2 and 3), indicating strikingly different expression profiles. Genes encoding ribosomal protein, protein synthesis factor (translation, ribosomal structure, and biogenesis), aminotransferase, spermine synthase, methionine synthase, glutamate-5-semialdehyde dehydrogenase, etc. (amino acid metabolism) were significantly upregulated in symbiotic *Serendipita* sp. compared to FLM but were not significantly differentially expressed in symbiotic *Tulasnella* sp. group (cluster 5). In addition, genes encoding rasGAP protein, Ca2+/calmodulin-dependent protein kinase, serine/threonine protein kinases (signal transduction mechanisms), sterol desaturase, and ergosterol biosynthesis protein (lipid metabolism) were upregulated in symbiotic *Serendipita* sp. compared to FLM. Notably, cluster 4 included 29 orthologous genes that were both upregulated in the two mycorrhizal fungi during interaction with *D. officinale* seeds in various symbiotic stages, especially in *Tulasnella* with the *D. officinale* seed group. These 29 genes primarily encode ubiquitin, short-chain dehydrogenase, ribosomal protein, histone, heat shock protein, glutathione S-transferase, carbohydrate-binding, ATPase, and amine oxidase, which are involved in carbohydrate metabolism, energy metabolism, and glutathione metabolism.

**Figure 7:**
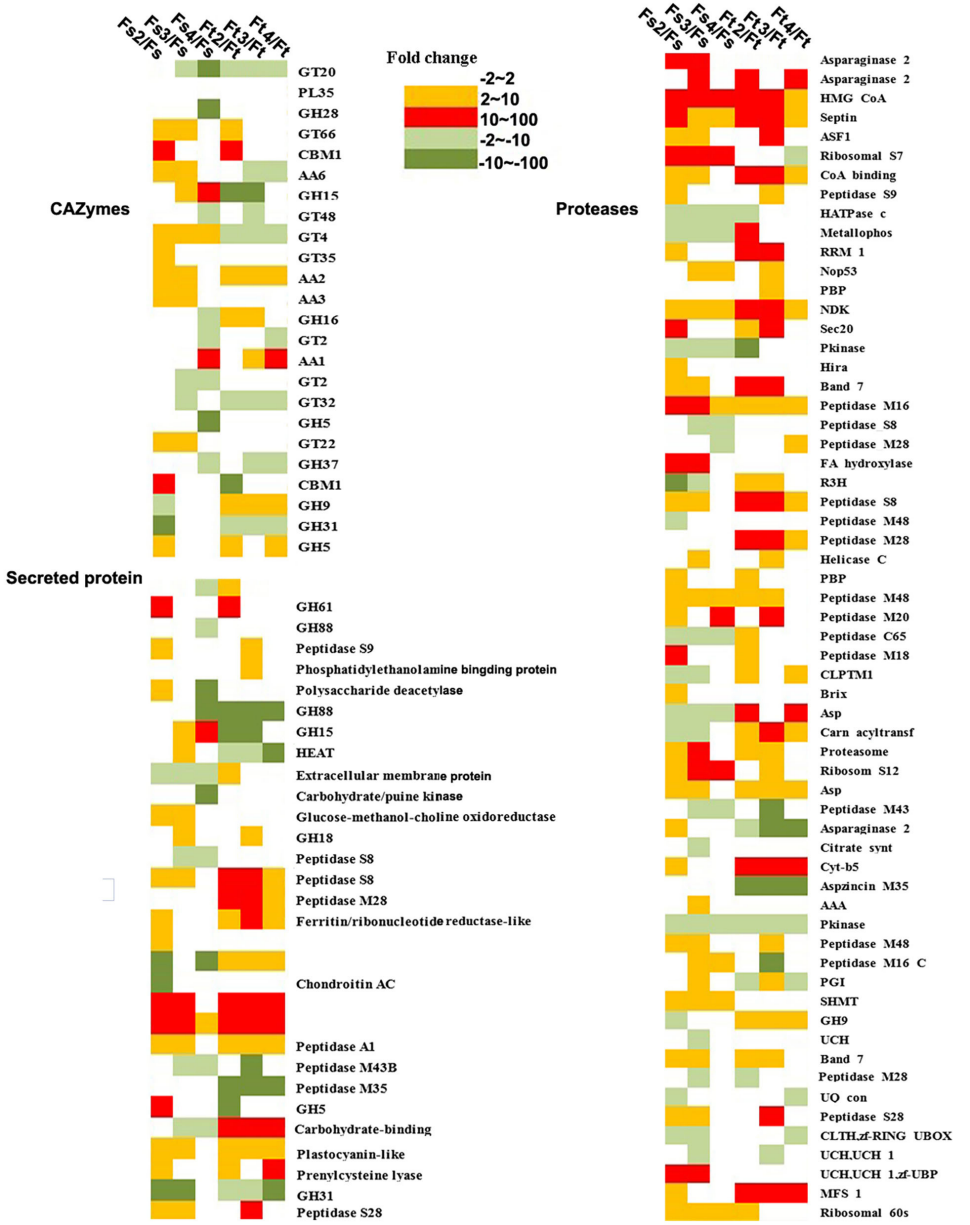
Heatmap showing the expression profile of fungal orthologous genes encoding CAZyme, secreted proteins, and protease during symbiotic germination of *D. officinale* with *Tulasnella* sp. and *Serendipita* sp. The expression level was evaluated using fold change in different colors. Green color (dark green means expression level with fold change ≥ 10) means genes down-regulated expression and red and orange color means genes up-regulated expression (red color means expression level with fold change ≥ 10). Ft2, Ft3, and Ft4 represent *Tulasnella* sp. in symbiotic statuses 2, 3, 4 stages while Fs2, Fs3, and Fs4 represent *Serendipita* sp. in symbiotic statuses 2, 3, and 4 stages (according to germination stage) and Ft and Fs means free-living mycelium of *Tulasnella* sp. and *Serendipita* sp., respectively.

To further understand the potential function of fungal orthologous genes during interaction with orchid seeds, we analyzed the fungal genes encoding CAZymes, protease, lipase, and SSPs, which were reported to likely be involved in mycorrhizal symbiosis in previous studies. The results revealed a total of 126 of 936 commonly expressed orthologous genes encoding the three specific protein categories (61 proteases, 24 CAZymes, and 31 SPs), and the expression profiles of the two symbiotic fungi during seed symbiotic germination of *D. officinale* at different stages (stage 2, stage 3, and stage 4) were analyzed (Figure 7). Twenty-four CAZyme genes were differentially regulated at the transcriptional level in at least one symbiotic stage compared to FLM. Genes encoding AA9 were both highly upregulated at the early germination stage (fungal invasion and peloton formation) after inoculation with *Tulasnella* sp. or *Serendipita* sp. with more than 100-fold changes. The gene encoding AA1 was upregulated during the seedling stage (fungal peloton digestion) in *D. officinale* with *Serendipita* sp. and *Tulasnella* sp. Genes encoding GT66 and GT4 and AA family proteins (AA2, AA3, and AA6) were upregulated during the early stage of germination and the protocorm formation of seeds with *Serendipita*. Genes encoding GH family proteins (GH16, GH9, and GH5) were markedly upregulated in at least one stage of seed symbiotic germination with *Tulasnella* sp. Among the 31 SPs, the genes encoding peptides S8, peptides M28, and carbohydrate-binding WSC domain proteins, representing secreted proteins, were specifically upregulated in the symbiotic *Tulasnella* group with more than a 100-fold-change compared to FLM. GH61 was upregulated in the initial invasion stages (or early germination stage) during symbiotic germination in the two symbiotic fungi, and GH5 was highly upregulated in symbiotic *Serendipita*.

In orthologous and commonly expressed gene sets, genes encoding proteases, such as asparaginase, HMG-CoA, septin, ASF, and peptidase, were significantly upregulated in at least one germination stage interaction in either *Tulasnella* sp. or *Serendipita* sp. Genes encoding cytochrome b5 and major facilitator superfamily proteins were specifically upregulated in symbiotic *Tulasnella* compared to FLM, with more than a 100-fold-change, while ribosomal S7 and fatty acid hydroxylase were highly upregulated in symbiotic *Serendipita* sp.

## Discussion

Orchid mycorrhizal symbionts of *Tulasnella* and *Serendipita* are the most common mycorrhizal partners of green orchids and belong to the phylum Basidiomycota. Here, we compared to plant and fungal gene expression in *D. officinale* seeds inoculated with two taxonomically different orchid mycorrhizal species (*Tulasnella* sp. S6 and *Serendipita* sp. 12825) at the transcriptional level. The two mycorrhizal fungi stimulated seed germination (stage 2), protocorm formation (stage 3), and seedling development (stage 4-5) in *D. officinale*.

### Expression of Genes Related to Plant Cell Wall Biosynthesis and Remodeling

Our comparative transcriptomics analysis revealed that an important proportion of plant genes (>60%) were expressed during the symbiotic germination of *D. officinale*. Most exhibited similar expression profiles at the same symbiotic germination stage with different mycorrhizal fungi. Genes encoding universal stress protein, UbiA, aquaporin-like, cytochrome P450 family, nodulin-like, plant lipid transfer protein; RAS domain family, and sugar (and other) transporter were significantly upregulated between the two mycorrhizal fungal combinations, indicating their common roles in the core mechanisms of symbiotic recognition, nutrition transport, or other aspects between the orchid host and the mycorrhizal fungi. These results are consistent with previous studies (Balestrini et al., 2014; Chen et al., 2017).

The plant cell wall is the headmost interface where interactions occur between the host plant and its mycobionts (Balestrini and Bonfante, 2014; Lionetti and Métraux, 2014). The plant cell wall is primarily composed of polysaccharides, such as cellulose, hemicelluloses, and pectins. In addition, there are some structural cell wall proteins characterized by highly repetitive sequence motifs, which are especially rich in specific amino acids, e.g., proline, glycine, and hydroxyproline glycoproteins, to modify the cell wall (Herger et al., 2019). In our present study, genes encoding epidermis-specific secreted glycoprotein, proline-rich receptor-like protein, leucine-rich repeat (LRR) extensin-like protein, and extensin-like protein were significantly upregulated during the symbiotic stage in *D. officinale* seeds after inoculation with *Tulasnella* sp. and *Serendipita* sp. Extensins are an abundant group of hydroxyproline-rich glycoproteins and are responsible for various processes, including embryonic development, root hair growth, cell wall assembly and structure, and biotic and abiotic stress responses (Castilleux et al., 2018). Most studies indicate that extensins exert their roles in plant defense by strengthening the cell wall, decreasing pathogen invasion, or facilitating the attachment of symbiotic organisms (Castilleux et al., 2021). In addition, new insights have been provided regarding the structure and role of extensins and their glycosylation in plant–microbe interactions using a set of anti-extensin-specific monoclonal antibodies (Castilleux et al., 2020), indicating that modeling of the cell wall architecture is likely to impact cell binding and pathogen colonization of roots. The extensins, especially arabinosylation, could even serve as markers of the plant immune response during plant-microbe interactions (Castilleux et al., 2018). In orchid symbiotic germination, previous studies revealed that the signals for the dynein monoclonal antibody JIM11 epitope were stronger in symbiotic *Dendrobium* protocorms than in asymbiotic ones and were primarily located in the plant walls and in special plant cells colonized by fungi rather than in the apical meristem. This indicated that the extensins were not only related to fungal attachment in a symbiotic relationship but were also likely critical for limiting fungal colonization in basal cells and preventing fungal spread inside the protocorms (Li et al., 2018). Regardless, our transcriptomics analysis indicated that the two mycorrhizal fungi both triggered plant genes encoding extensions to be significantly upregulated in the symbiotic stage during interaction with *D. officinale* seeds, consistent with the results of previous studies and further provided supported data.

Immunofluorescence labeling revealed that microtubules were altered and reorganized adjacent to the symbiosis interface after the fungus invaded root cells (Genre and Bonfante, 2002), although the ultrastructural changes were not illustrated in detail in the orchid mycorrhiza. In our study, the gene encoding microtubule-associated protein was upregulated in the symbiotic stage, indicating that cytoskeletal rearrangements likely occurred during the invasion of orchid seeds by the fungus. In addition, genes related to plant cell biosyntheses, such as those encoding cellulose synthase and pectinesterase, were significantly upregulated when inoculated with either *Tulasnella* sp. or *Serendipita* sp. compared to asymbiotic germination, indicating that plant cell wall biosynthesis and reinforcement was very active during the interaction between the mycorrhizal fungi and *D. officinale* seeds.

### Fungal Gene Expression Related to Plant Cell Wall Degradation Enzymes

During interaction with the same host plant, *Tulasnella* sp. induced more genes involved in the symbiotic germination of *D. officinale* than did *Serendipita* sp. For example, during the initial invasion stage (correspondingly, the early germination stage), a total of 8,128 fungal genes in *Tulasnella* (5,268 up- and 2,960 downregulated) were differentially expressed compared to FLM, while there were 4,630 differentially expressed genes in *Serendipita* sp. (2,066 up to and 2,564 down). Although no genome information is available for either mycorrhizal fungus, using an overview of the reported genome of *Tulasnella calospora* (62.39 Mb, 19,635 genes) and *Serendipita vermifera* (38.09 Mb, 15,317 genes) (Kohler et al., 2015), we suspect that different taxon of mycorrhizal fungi could be contributed to the gene expression difference during interaction with the orchid plant to the extent.

In addition, we identified 4,954 orthologous genes based on the transcriptome of *Serendipita* sp. and *Tulasnella* sp. A total of 1,682 genes were expressed in symbiotic *Serendipita* sp. and 2,464 genes were expressed in symbiotic *Tulasnella* sp. compared to the FLM during interaction with *D. officinale* seeds (in at least a symbiotic condition). A total of 936 orthologous genes were commonly expressed, and most of the genes exhibited similar expression during mycorrhizal interactions with any mycobiont, suggesting that different mycorrhizal fungi shared a common set of genes involved in symbiotic responses.

### CAZymes

Genome analysis revealed that orchid mycorrhizal symbionts had the largest set of CAZymes supporting their dual saprotrophic/symbiotic lifestyles (Martin et al., 2016). For example, *T. calospora* has 7 GH6, 27 GH7, and 33 LPMO genes for the degradation of crystalline cellulose (Kohler et al., 2015). Proteins with a cellulose-binding domain (CBM1) are also abundant in orchid mycorrhizal fungi (ORM). In our present study, 66 DEGs encoding CAZymes were identified in symbiotic *Tulasnella* sp., 99 genes encoding CAZymes were differentially expressed in symbiotic *Serendipita* sp., and AA family members, CBM, CE, and GH, were upregulated in symbiotic fungi compared with FLM. CBM 43 (β-1,3-glucanosyltransferase, former X8) and CBM48 (starch debranching enzymes) are not common in the known various fungal lifestyles, and their exact function in orchid mycorrhizal fungi is also unclear, but their significantly high expression in symbiotic *Tulasnella* sp. implies that they likely play a vital role in fungal cell wall construction because they are required for fungal cell wall component dual β-(1,3)-glucan elongation and branching (Aimanianda et al., 2017). In addition, chitin deacetylases of the CE4 family were upregulated in symbiotic *Tulasnella* sp. and chitosan, the product of *Tulasnella* chitin deacetylases has been identified by metabolomic analyses in symbiotic orchid protocorms (Ghirardo et al., 2020). The role of CE4 is suspected to protect the fungal cell wall from acting by plant chitin or decreasing the plant defense responses when the fungal colonization transfers chitin to chitosan in the ECM (Veneault-Fourrey et al., 2014).

Genes encoding the lignin-degrading auxiliary enzyme AA class were also upregulated in symbiotic *Tulasnella* sp. and *Serendipita* compared to FLM, especially AA9, a family of fungal origin with only lytic polysaccharide monooxygenases (Vandhana et al., 2021). AA9 is a mono-copper enzyme family and participates in the degradation of lignocellulose via the oxidative cleavage of celluloses, cello-oligosaccharides, or hemicelluloses (Zhang, 2020). Zarattini et al. (2021) employed AA9 LPMO reaction products to trigger innate immune responses in the model plant *Arabidopsis thaliana* during fungal pathogenesis, while results on AA17 LPMO indicated that LPMO action would help overcome plant defense barriers (Sabbadin et al., 2021). Thus, the role of AA9 in orchid symbiotic germination needs to be further investigated. In addition, the glycoside hydrolase family GH5 and GH28 were recently confirmed to be involved in cell wall remodeling for ECM symbiosis as enzymatic effectors (Zhang et al., 2021). In our study, the expression of many glycoside hydrolase family members was induced in symbiotic tissue, such as genes encoding the hemicellulose-active enzyme GH3 (β-glucosidases), which were both upregulated in the symbiotic condition of the two fungi, indicating that they likely play a key role in orchid mycorrhizal establishment and development.

Of note, several genes encoding glycosyltransferases (GTs) were differentially expressed in symbiotic *Tulasnella* sp. or *Serendipita* sp. mycelium, and there were 9 GT family proteins (GT2, GT4, GT20, GT22, GT32, GT35, GT48, GT66) with induced expression among the 24 orthologous genes encoding CAZymes. Glycosyltransferases are enzymes that catalyze the transfer of sugar moieties from activating donor molecules to specific acceptor molecules, forming glycosidic bonds and are involved in the biosynthesis of diverse carbohydrates (http://www.cazy.org/GlycosylTransferase-family). A recent result indicated that GT2 likely participates in the synthesis of extracellular or outer cell wall polysaccharides, which play a key role in facilitating many interactions between plants and fungi by enabling hyphal growth on solid matrices (King et al., 2017). However, the exact function of glycosyltransferases in orchid mycorrhizae still needs to be characterized. Adamo et al. (2020) primarily explored the roles of CAZymes of orchid mycorrhizal fungi in the development of unsuccessful and successful interactions and suspected that the expression of some key CAZymes was related to OMF switches from symbiotic to saprotrophic growth. However, the mycorrhizal relationship is particularly complex in orchids, and successful mycrrhizal interactions between orchids and their symbionts can be controlled by a balance between fungal invasion and plant defense, balancing nutritional supply and demand; therefore, the specific nutritional strategy of orchids should be considered for mycorrhizal establishment.

In addition, *Tulasnella* and *Serendipita* possessed several CAZyme, but they display different expression profiles, indicating that their function could be different in fungal species. Moreover, only a few orthologous genes encoding CAZymes were identified. Among the 24 orthologous DEGs encoding CAZymes, most exhibited distinct expression profiles between *Tulasnella* and *Serendipita*, and only genes encoding GH5, AA1, AA2, CBM1, and GT66 were upregulated in at least one symbiotic stage in both *Tulasnella* and *Serendipita*. Thus, about function of CAZymes in different mycobionts during interaction with the host plant still need to be addressed furtherly in the future.

## Conclusion

A high proportion of plant genes were induced, and most genes displayed similar expression profiles in *D. officinale* seeds inoculated with the two different fungal species of *Tulasnella* sp. and *Serendipita* sp., indicating that plant genes participate in symbiotic relationships, which might be conserved in orchid mycorrhizae. We also highlight the differences and similarities in fungal gene expression between *Tulasnella* sp. and *Serendipita* sp. during interactions with the same host plant. Comparative transcriptomics analyses identified a core set of orthologous genes between *Tulasnella* sp. and *Serendipita* sp. involved in symbiotic orchid germination. Most symbiosis-induced genes are restricted to a single fungal species. Several genes encoding CAZyme likely contribute to establishing orchid mycorrhizal interactions. The available transcriptome sequences of mycorrhizal fungi interactions with orchid plants represent foundational information for a better understanding of symbiosis development, the function of orchid mycorrhiza, and orchid seed biology.

## Data Availability Statement

The datasets presented in this study can be found in online repositories. The names of the repository/repositories and accession number(s) can be found below: National Center for Biotechnology Information (NCBI). SRA database BioProject database under accession number PRJNA805043.

## Author Contributions

JC, FM, and SG designed the experiments. YT and DZ performed the experiments. JC, YT, AK, AL, YL, and YX analyzed the data. FM discussed the result and gave great suggestions. JC wrote the manuscript draft and all authors revised and discussed it. All authors contributed to the article and approved the submitted version.

## Funding

This work was funded by the National Natural Science Foundation of China (81973423) and the CAMS Innovation Fund for Medical Sciences (CIFMS) (2021-I2M-1-032).

## Conflict of Interest

The authors declare that the research was conducted in the absence of any commercial or financial relationships that could be construed as a potential conflict of interest.

## Publisher's Note

All claims expressed in this article are solely those of the authors and do not necessarily represent those of their affiliated organizations, or those of the publisher, the editors and the reviewers. Any product that may be evaluated in this article, or claim that may be made by its manufacturer, is not guaranteed or endorsed by the publisher.

## Supplementary Material

Supplementary Figure S1 Venn diagram showing the number of fungal genes differentially expressed across various symbiotic stages of *D. officinale* inoculated with *Tulasnella* sp. and *Serendipita* sp. **(A–D)**. **(E)** Functional classification of 2,555 co-upregulated fungal genes of *Tulasnella* sp. in symbiotic status compared to free living mycelium; **(F)** Functional classfication of 403 co-upregulated fungal genes of *Serendipita* sp. in symbiotic status compared to free-living mycelium.

Supplementary Figure S2 Cluster analysis of 936 fungal orthologous differentially expressed genes. All genes analyzed were divided into five clusters. F_S2, F_S3, F_S3 means *Serendipita* sp. in symbiotic statuses 2, 3, and 4 stages, and F_T2, F_T3, and F_T4 means *Tulasnella* sp. in symbiotic statuses 2, 3, and4 stage (according to seed germination), Ft and Fs means free-living mycelium of *Tulasnella* sp. and *Serendipita* sp., respectively.

Supplementary Table S1 Functional classification of 452 upregulated expressed plant genes across various symbiotic germination stages of *D.officinale* inoculated with *Tulasnella* sp. or *Serendipita* sp.

Supplementary Table S2 25 most up-regulated common expression plant genes across germination stage whatever inoculated with *Tulasnella* sp. and *Serendipita* sp. (Fold change ≥ 5).

Supplementary Table S3 The KEGG pathway annotation of 936 orthologous expressed genes in 5 clusters.
