# Supplementary figures and images for: Comparative Transcriptomics Analysis of the Symbiotic Germination of D. officinale (Orchidaceae) With Emphasis on Plant Cell Wall Modification and Cell Wall-Degrading Enzymes
Source: Front Plant Sci. 2022 May 6;13:880600. doi: 10.3389/fpls.2022.880600 (PMC9120867; doi:10.3389/fpls.2022.880600)

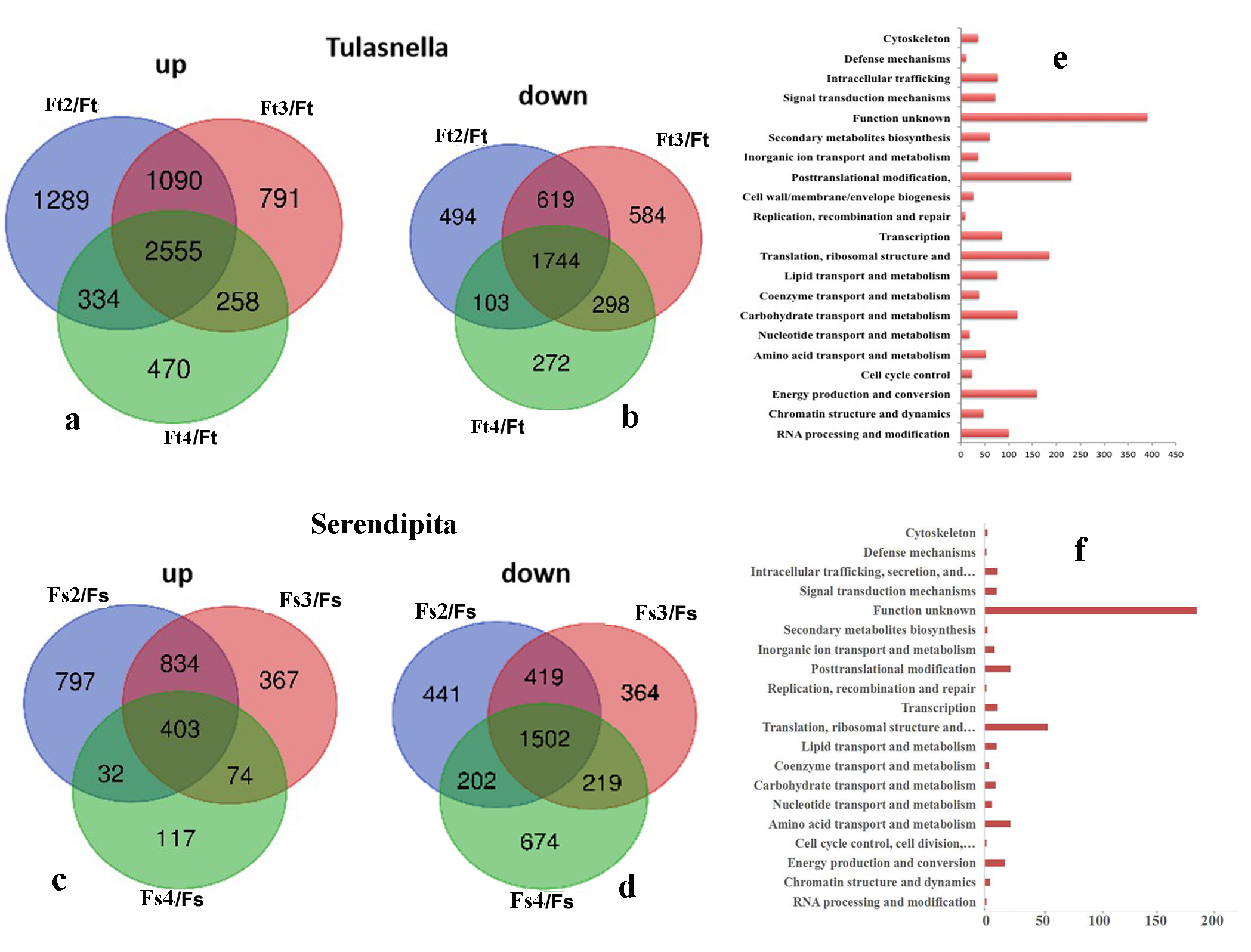

Supplement: Supplementary Figure S1 — Venn diagram showing the number of fungal genes differentially expressed across various symbiotic stages of D. officinale inoculated with Tulasnella sp. and Serendipita sp. (A–D). (E) Functional classification of 2,555 co-upregulated fungal genes of Tulasnella sp. in symbiotic status compared to free living mycelium; (F) Functional classfication of 403 co-upregulated fungal genes of Serendipita sp. in symbiotic status compared to free-living mycelium. [file Image_1.JPEG]

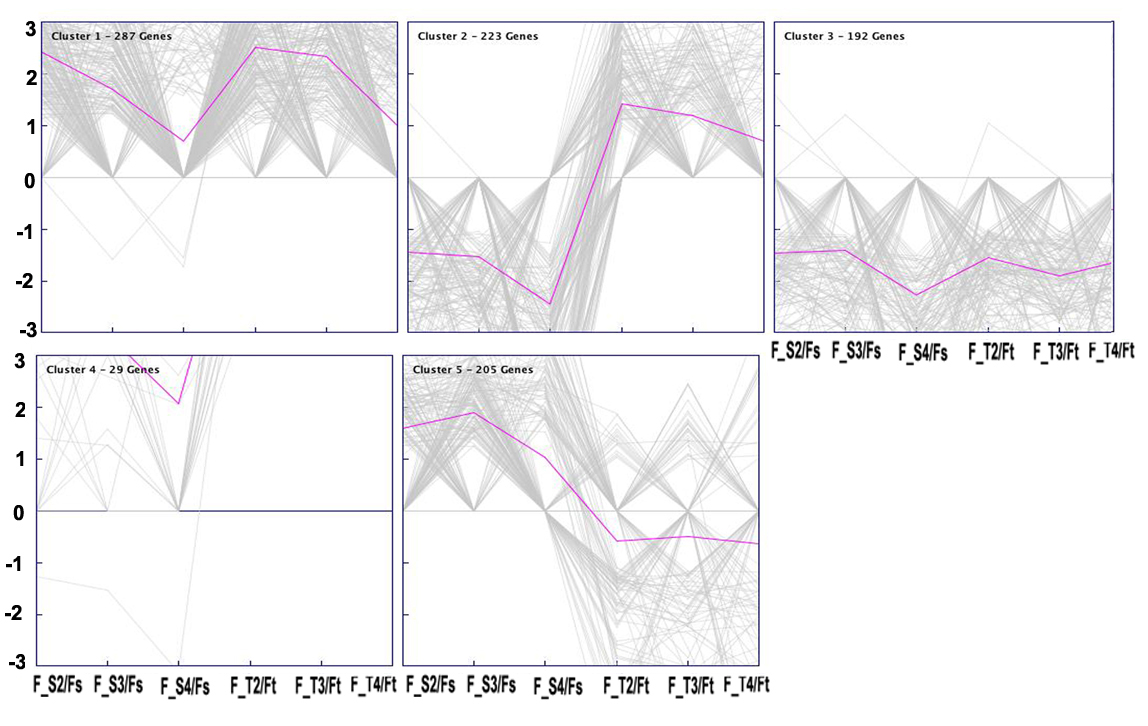

Supplement: Supplementary Figure S2 — Cluster analysis of 936 fungal orthologous differentially expressed genes. All genes analyzed were divided into five clusters. F_S2, F_S3, F_S3 means Serendipita sp. in symbiotic statuses 2, 3, and 4 stages, and F_T2, F_T3, and F_T4 means Tulasnella sp. in symbiotic statuses 2, 3, and4 stage (according to seed germination), Ft and Fs means free-living mycelium of Tulasnella sp. and Serendipita sp., respectively. [file Image_2.JPEG]
